# Interprofessional Dementia Education in Pre‐Registration Healthcare Students: A Systematic Review

**DOI:** 10.1002/gps.70213

**Published:** 2026-05-16

**Authors:** Molly Hebditch, Stephanie Daley, Pamela Rae, Susie Muszynska, Yvonne Feeney, Sube Banerjee

**Affiliations:** ^1^ Centre for Dementia Studies Brighton and Sussex Medical School Trafford Centre University of Sussex Brighton East Sussex UK; ^2^ Plymouth Integrative Health and Social Care Education Centre (PIHC) Faculty of Health University of Plymouth Plymouth UK; ^3^ Centre for Dementia Studies Sussex Partnership NHS Foundation Trust Trafford Centre University of Sussex Brighton East Sussex UK; ^4^ Faculty of Medicine and Health Sciences University of Nottingham Nottingham UK

**Keywords:** dementia, healthcare students, interprofessional education, pre‐registration, undergraduate

## Abstract

**Objective:**

High‐quality dementia care is underpinned by interprofessional collaborative practise, and healthcare training is a critical opportunity to develop these skills. This review aims to examine the evidence for whether interprofessional dementia education for undergraduate healthcare students positively impacts outcomes related to collaborative practise.

**Methods:**

Inclusion criteria consisted of papers investigating dementia interprofessional education interventions delivered within undergraduate education and that assessed outcomes relating to interprofessional collaboration. Searches were limited to papers published after 2014 and were conducted in eight databases: PubMed/MEDLINE, EMBASE, Web of Science, ERIC, The Cochrane Library, PsycINFO, CINAHL, Applied Social Sciences Index and Abstracts (ASSIA), British Education Index (BEI). A narrative synthesis was conducted and data quality was assessed.

**Results:**

11 papers, evaluating 11 different interventions, were included in the narrative synthesis. Four studies demonstrated positive changes in student attitudes or perceptions of interprofessional education or collaboration. Seven studies had evidence that students' knowledge or perceived skills about interprofessional collaboration increased. No evidence was presented for change in student behaviour, or impact on patients or organisational practise. Three studies presented findings on outcomes specifically relating to interprofessional dementia care.

**Conclusions:**

The findings suggest that dementia interprofessional education may contribute to interprofessional competencies. Therefore, interprofessional education could provide added value to dementia education, both of which are high priorities in the undergraduate curriculum. However, the strength of the evidence was weak as the methodological quality of the papers was low. Additionally, interventions were varied and therefore, optimal components of dementia interprofessional education were not identified. More rigorous investigation is needed on the impact of dementia interprofessional education with a focus on the longer‐term impact on student practise and service delivery.

## Key Points

- This is the first systematic review to assess the evidence on whether dementia interprofessional education can improve outcomes related to interprofessional collaboration in undergraduate healthcare students.
- This paper identified 11 dementia interprofessional education interventions that indicate some evidence that they can improve student outcomes in relation to attitudes towards interprofessional education or collaboration, or improve knowledge or skills in interprofessional collaboration.
- The strength of evidence is limited due to poor methodological design, including a lack of control groups.

## 1 Introduction

The core of current and future quality health service delivery is teamwork and so, interprofessional education (IPE), where students from two or more professions ‘learn with, from and about each other’ [1], is a vital component of undergraduate healthcare curricula to develop collaborative practise [2, 3, 4]. Collaborative practise is essential in modern‐day healthcare as multidisciplinary services are pivotal in managing the increases in chronic conditions, an ageing population and high multimorbidity [4, 5]. Dementia is an exemplar of a complex condition that requires effective interprofessional collaboration (IPC) [6, 7, 8] and, therefore, is fertile ground for interprofessional education. However, the content and outcomes of such education and optimisation of delivery are not well evidenced.

Jackson et al., [9] in 2016, conducted a systematic review of interprofessional education in the care of people with dementia. The review aimed to identify, describe and evaluate the impact of IPE interventions on health and social care practitioners' (pre‐qualification and post‐qualification) understanding of dementia, the quality of care for people with dementia, and support for their carers. Six studies met their criteria, only two of which investigated students [10, 11]. Outcomes were evaluated against a modified Kirkpatrick's model, developed by Barr et al., [12] which has been previously used in evaluations of IPE [2, 13, 14], and support for lower‐level outcomes (attitudes and knowledge) was found. However, no studies reported any evidence of impact on student behaviour, organisational delivery, or patient outcomes. In the decade since that review, there has been a notable increase in dementia‐specific educational initiatives at the undergraduate level [15]; therefore, a new systematic review is of potential value.

This review explores the outcomes of dementia IPE interventions delivered in undergraduate curricula. Specifically, this review explores outcomes related to interprofessional collaboration. Whilst recent systematic reviews have investigated student outcomes for dementia education interventions, and have identified dementia IPE interventions within this [15, 16], the evidence of these interventions on the impact on IPC has not been explored. This review also distinguishes between dementia‐specific IPC outcomes versus generic IPC outcomes. IPC is a general skill which also has specific considerations for dementia care. Therefore, it is crucial to understand whether dementia IPE interventions are impacting skills and perceptions related to general IPC, or IPC within the context of dementia care.

### 1.1 Review Question

This systematic review aims to examine the evidence for whether interprofessional dementia education for undergraduate healthcare students positively impacts outcomes related to collaborative practise.

Research questions:

1. Does IPE in undergraduate dementia education impact student attitudes, knowledge, and behaviour in relation to generic IPE/IPC?
2. Does IPE in undergraduate dementia education impact student attitudes, knowledge, and behaviour in relation to dementia‐specific IPE/IPC?
3. Have studies investigated longer‐term organisational practise or patient outcomes? If so what have they found?

## 2 Method

The protocol for this review was registered on PROSPERO [ref: CRD42024552308] and PRISMA reporting guidelines have been followed [17] (See Appendix A).

### 2.1 Eligibility Criteria

The inclusion criteria for topics of papers are summarised in Table 1. The criteria for types of papers included empirical peer‐reviewed primary research papers (including theses), with no restriction on study design. Studies that did not report primary empirical findings (i.e., systematic reviews, protocol papers) or were not peer‐reviewed (i.e., abstract proceedings, reports) were excluded.

**TABLE 1:** Eligibility criteria.

Population/Participants of interest:	Inclusion: Undergraduate healthcare students People with dementia/family carers Organisation (outcomes) Exclusion: Non‐healthcare exclusively, post‐registration/staff
Interventions or exposures	Inclusion: IPE pre‐registration dementia education IPE: Must include elements of two student groups learning together. Pre‐registration students; Dementia education; Education can take all formats; lectures, placements, online courses, and conferences. Exclusion: Interventions delivered to staff, postgraduate students, and education related to older adults (not dementia‐specific content)
Comparator(s)/control	No restrictions on comparison groups.
Outcomes	Any outcomes relating to IPE or IPC (dementia specific or generic). These could relate to one of five outcome levels [12]: Modification of attitudes/perceptions (students) Acquisition of knowledge/skills (students) Behavioural change (students) Change in organisational practise (organisation) Benefits to patients/clients (people with dementia/carers). Outcomes can be quantitative or qualitative. Additional outcomes: Where outcomes have been included for organisations or patients, all outcomes will be included. Exclusion: Outcomes only related to general dementia attitudes, knowledge or behaviours (not IPE related).
Study design	Any quantitative, qualitative or mixed‐methods study No restriction on design.

### 2.2 Information Sources

Articles were identified through systematic searches of the following electronic databases: PubMed/MEDLINE, EMBASE, Web of Science, ERIC, The Cochrane Library, PsycINFO, CINAHL, Applied Social Sciences Index and Abstracts (ASSIA), British Education Index (BEI). Searches were conducted on 25.06.2024. Searches included papers published after 2014; to include 10 years not previously examined in a previous systematic review [9]. Only studies published in the English language were included. In addition, further searches were conducted on 29.04.25, which included reviewing the references of included studies (snowballing), and searching for papers using the ‘related’ and ‘cited by’ functions of online databases (lateral searching) for included studies and previous review.

#### 2.2.1 Search Strategy

The search strategy was based on three components relating to the intervention of interest: (i) Interprofessional education (IPE), (ii) dementia, and (iii) students/pre‐registration; it was informed by a review of relevant literature known to the authors. The full search strategy is presented in Appendix B.

#### 2.2.2 Selection Process

##### 2.2.2.1 Duplicate removal

All studies identified from searches were entered into an electronic database and duplicates were removed.

##### 2.2.2.2 Initial Screening

AI was used to screen titles and abstracts using ASReview [18], to reduce the time burden of screening. The SAFE procedure was followed, which was developed to provide guidance and transparency around the use of screening with AI [19]. First, the research team met to predefine the stopping criterion and create a checklist of eligibility criteria to aid screening. Next, the initial pre‐screening to provide training data was completed by the team (MH, SM, PR, YF) before active learning was applied (MH & SM). The process of screening using the SAFE procedure, is documented in Appendix C.

##### 2.2.2.3 Full‐Text Screening

Full texts were retrieved and added to an electronic database [20], and then screened by two reviewers independently (MH & SM or PR or YF), and disagreements were resolved by discussion.

#### 2.2.3 Data Extraction

A data extraction form was developed and piloted. The form included details for evidence synthesis and quality assessment.

- Publication details: author, year of publication, title, country, aims/objectives,
- Methods: study design, comparison/control group, sample size, duration, study setting (university),
- Participant characteristics: Students: demographics, student group. Organisation: all details provided. Patients: demographics and settings.
- Characteristics of intervention: intervention components, intended outcomes, setting, and delivery mechanisms.
- Outcomes: measures, author's definition of relevant constructs (e.g., Attitudes to IPE or knowledge of IPC), findings (quantitative analysis: effect measure, significance and association, and qualitative analysis: main themes).

For synthesis, two reviewers independently categorised each quantitative outcome (measure) or qualitative outcome (theme) by outcome level (described in Table 1). Outcome levels were based on a modified Barr et al., framework [2, 12] and identified whether IPE outcomes were dementia‐related or generic. All discrepancies were identified and agreed upon. Also, the main teaching modalities of each intervention were labelled. This included didactic teaching, simulation, experiential, reflective activities and problem‐based learning (PBL) or case‐based learning (CBL) [14].

### 2.3 Risk of Bias

Risk of bias was quantified using the Mixed Methods Appraisal Criteria (MMAT) [21]. If papers included multiple methods for different outcome measures, only methods relating to IPE outcomes were reviewed. Each paper was quality‐rated by two independent reviewers; disagreements were discussed with a third reviewer. Studies were not excluded based on ratings due to limited literature; this review was exploratory and assessed the overall quality of current literature.

### 2.4 Synthesis

A narrative summary was produced alongside tabular representations of findings for study characteristics, educational interventions, and outcome measures. An overview of educational interventions is presented, and a summary of the strength of evidence for each level of learning outcomes. Findings related to dementia‐specific and generic outcomes are identified. A quantitative synthesis or meta‐analysis was not appropriate due to heterogeneous study designs.

## 3 Results

### 3.1 Study Selection

**FIGURE 1:**
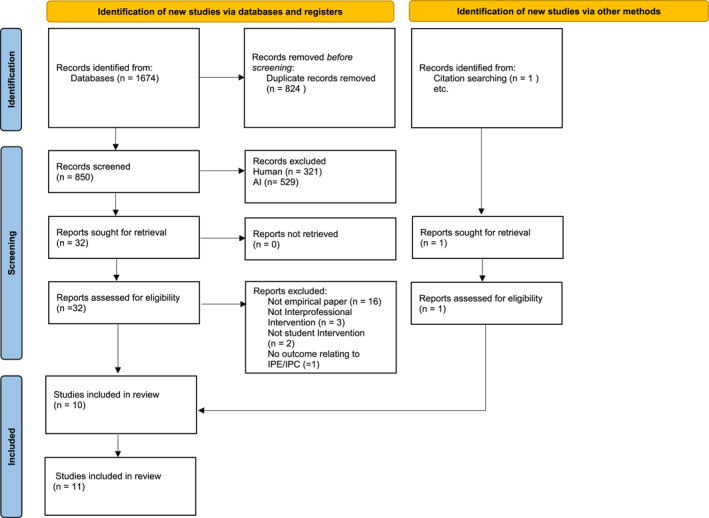
Flow diagram of study selection.

We identified 1674 studies in the database search. After duplicates were removed, 850 studies were screened, from which 32 full texts were screened. 11 papers were assessed as relevant, including the addition of one paper found in additional searches [22]. A flow diagram of study selection is presented in Figure 1.

### 3.2 Interventions Characteristics

An overview of IPE interventions evaluated in these studies is presented in Table 2. Interventions evaluated were mostly from Australia (*n* = 3) [10, 23, 27] and the USA (*n* = 3) [28, 30, 31], two were conducted in Germany [26, 29] and the UK [24, 25], and a single intervention was from Ireland [22]. Most interventions included three or more student types (*n* = 8), with one study having 11 disciplines taking part in the intervention and evaluation [25]. Eight of the studies described anticipated student learning outcomes for the intervention; of these, six mentioned outcomes related to IPE.

**TABLE 2:** IPE interventions.

Study author Country	Intervention name	Student Types	Type of intervention Description	Duration	Learning outcomes described? Yes/No: Detail	Evidence of IPE outcomes Quant/Qual: Outcome level^a^—Description
Annear et al. [23] Australia	Wicking dementia research and education centre (WDREC) teaching aged care facilities (TACF) programme	Medical, nursing, and paramedic students	CBL (clinical setting) A seven‐step protocol that includes collaborative assessments of older people in a dementia care setting and the development of collaborative care plans.	Five‐day interprofessional clinical placements	Yes: attitudes towards IPE and knowledge of dementia.	Quant: Level 2—Improved communication and teamwork, and interprofessional relationships
Dingwall et al. [24] UK	Sliding doors	Nursing and social work students	Reflective activity Elements of interactive drama‐based learning, theatre, and participatory learning. A drama between an older person with early dementia and her husband is played out by actors and used to stimulate discussions on what makes a ‘good life’. This is situated in the context of practise implications current policy and practise landscape, service delivery system and on a personal practise level.	One‐day workshop	Yes: Seven outcomes described, including explicit IPE outcomes e.g ‘recognise how to work together more effectively’	Quant: Level 1—positive and negative changes in attitudes towards IPC (social work students only) Qual: Level 1—Positive attitudes to interprofessional working; however, there was also evidence of negative perceptions of other student groups
Davison, et al. [25] UK	DA learning package	Medicine Nursing Speech and language therapy Occupational Therapy Physiotherapy Pharmacy Paramedic science	Didactic and reflective activity One‐hour dementia friends session (face‐to‐face in an interprofessional group, or online). Video on family members sharing experiences of dementia. Reflective student workbook. Multiple‐choice‐questionnaire 10 questions (8/10 TO pass).	Unclear duration	Yes: In relation to Tier 1 dementia awareness (DA) qualification‐ dementia learning outcomes only	Qual: Level 2—Positive accounts of learning to work together
Dressel, et al. [26] Germany	Interprofessional dementia workshop	Physiotherapy, nutrition therapy and counselling and speech‐language pathology	Didactic and simulation Workshops taught by experienced staff on three themes (covered each day): (1) Understanding of roles. (2) Collaborative dementia management. (3) simulated interprofessional case‐conference with role‐play and development of a multicomponent treatment approach.	Three‐day workshop, break between second and third day	Yes: Objectives related to generic competencies in knowledge and skills about dementia and dementia management and dementia‐specific and general competencies for interprofessional collaboration (table of learning objectives presented)	Quant: Level 2—Improved communication and teamwork, interprofessional relationships and attitudes to interprofessional learning. Level 2—Improved knowledge and skills related to interprofessional teamwork in dementia management
Lawlis, et al. [27] Australia	IPE residential aged care facilities (RACF) programme	Nursing, Occupational therapy,and aged care diploma	CBL (clinical setting) The students were allocated to two groups of 6 (comprising two students from each profession) and assigned a project with RACF supporting nutrition (‘dementia friendly eating tools’ or ‘food memories’); weeks 1 and 2 were designed so that students either observed or interacted with the residents, with the intervention implemented during week 3 followed by a formal discussion with educators.	Three‐weeks (3h per week, 9h in total)	No	Quant: Level 1—Improved attitudes to IPE Qual: Level 2—Positive accounts of IPE including teamwork skills and knowledge of other professionals
Mastel‐Smith, et al. [28] USA	Dementia care boot camp	Nursing, occupational therapy assistant, pharmacy and psychology	Didactic, simulation and PBL In interprofessional groups, multiple content in teaching and group work. The content was delivered via various learning methods: Teepa snow, a dementia care expert and trainer, videos in which dementia care was demonstrated; case‐specific vignettes; role play; and a dementia simulation, the virtual dementia Tour (VDT), and talks on lived experiences by person with dementia. Activities such as case studies and role play were completed in teams.	16 h (two, eight‐hour sessions) in one semester	Yes: 16 learning outcomes described and included promote students' knowledge, attitudes, empathy, and self‐confidence for dementia care, one related to IPE : Learn with, from and about other how students from other disciplines assess, intervene, and evaluate care of people with dementia.	Qual: Level 1—positive accounts of IPE intervention
Balzer, et al. [29] Germany	Better COMpetencies for the inter‐professional and individually appropriate care for people with DEMentia (KOMPIDEM)	Medical and nursing students	Didactic, PBL (theoretical) and experiential Four components: Introductory lectures to ensure a common basic knowledge level amongst all participants. Problem‐based learning (PbL) in small groups to pro‐mote a more detailed and activity‐oriented engagement of the students with certain aspects of dementia care as well as inter‐professional communication. Visitations to care facilities to promote students' awareness of existing challenges and problem‐solving strategies applied in the care for people with dementia. A final colloquium comprising presentations by the PBL groups.	30 h (contact hours of the participants with lecturers, tutors or healthcare representative)	Yes: 3 core objectives described regarding dementia knowledge and skills and one related to IPE: awareness of the own professions and the other professions roles and responsibilities in dementia care	Quant: Level 1—Positive appraisal of IPE intervention
Brown et al. [30] USA	Novel geriatric simulation‐enhanced interprofessional education (gero IPE‐sim)	Nursing, nutrition, social work, speech language pathology (SPL)	Simulation Six components: (1) pre‐simulation learning, (2) pre‐briefing review of IPCP competencies, (3) simulation role‐play with debriefing, (4) breakout learning sessions, (5) daily session debrief, and (6) post‐education activities with reflection discussion cues and daily evaluation surveys.	Six hours in person ‐ 2 h sessions on three different days across 3 weeks.	No (however, students were oriented to the interprofessional education collaborative core competencies (2016))	Quant: Level 1—positive appraisal of IPE learning outcomes, level 2—Reduction in perceived challenges of IPC
Cartwright et al. [10] Australia	No name: ‘Online dementia case study'	Speech pathology, health information management Social work students, occupational therapy, nursing students	CBL (theoretical) The online case study ran over a 4‐week period, in interprofessional student groups, each with a facilitator. Authentic clinical resources included extracts of inpatient case notes, footage of staff interactions on the ward, medical reports and test results.	Four weeks, (time unknown)	Yes: Increase awareness of the complexities and challenges that characterise dementia care, whilst demonstrating the value of collaborative, client‐centred practisce (specific list of objectives provided)	Quant: Level 2—Increases in students' ability, value and comfort in working with others Qual: Level 1—accounts of improved attitudes to IPC
Chambers et al. [31] USA	No name: ‘Simulation and education module about delirium'	Nursing & medical students	Simulation The simulation involved two actors: a Standardized pa‐patient with chronic dementia who recently developed symptoms of delirium and a standardized family member who brought the patient to the emergency department. The debrief focussed on appropriate assessment and treatment of delirium, communication including check‐back and call out as defined by team STEPPS (AHRQ, 2013), reflection on roles and responsibilities and teamwork.	20 min simulation, 30 min debrief	Yes: ‘recognize delirium in a patient with chronic dementia, to foster effective communication between medical and nursing students, to understand respective roles and responsibilities, and to use teamwork to provide care’	Quant: Level 1—Positive change in perceptions of and attitudes towards IPE, teamwork, and simulation.
O'Sullivan et al. [22] Ireland	No name: ‘IPL dementia workshop'	Medicine, nursing, dentistry, physiotherapy, radiography, radiation therapy, audiology, speech and language therapy, pharmacy, occupational therapy, and paramedicine	CBL (theoretical) The workshop started with a patient advocate presentation. (20 min), followed by a dementia presentation from a medical professional (45 min). A dementia case study is introduced which is followed by interprofessional group work. (1.5 h), which is followed by a Q & A session with expert panel from each discipline.	Approx. 3 h	No	Quant: Level 1—Increased awareness of the roles of other healthcare professions Qual: Level 2—Accounts of improved interprofessional communication and understanding others' roles

^a^ based on a modified Barr et al. framework [12].

Interventions were varied and often comprised multiple modalities, especially for longer‐duration interventions. Five interventions had a duration of less than one day. The teaching modalities of these included simulations (*n* = 2) [30, 31], didactic and reflective activity (*n* = 1) [25], CBL (*n* = 1) [22] and a novel reflective activity (*n* = 1) [24]. Six interventions had a duration of 8 h or more, with the longest being a 5‐day interprofessional clinical placement [23]. The modalities of these interventions included CBL (*n* = 3) [10, 23, 27], didactic, PBL (theoretical) and experiential (*n* = 1) [29], simulation and didactic (*n* = 1) [26], didactic, simulation and PBL (*n* = 1) [28]. All 11 studies presented some evidence for positive IPE outcomes; therefore, no teaching modality or duration of intervention could be identified as having more evidence.

### 3.3 IPE Outcomes

IPE outcomes and methodological details for each study are presented in Table 3.

**TABLE 3:** Evidence of IPE outcomes.

Study author MMAT score*/***** [Overall design—components]						
Quantitative (Quant): Measures	Outcome (construct)	*N* (% follow‐up response rate, if relevant)	Data collection method	Results (IPE outcomes only extracted) Significance (*p*‐value), association (+/−/not significant NS) [sub scales/sub analysis]	Dementia‐related IPE outcomes (Yes/No)	Evidence of outcome level^a^ (1–5)
Qualitative (Qual): Type of data	Topic		Analysis	Themes & description (Only IPE related themes presented)		
Studies with level 1 evidence: Modification of attitudes and perceptions						
Dingwall, et al. [24].** [mixed methods ‐ pre and post survey with focus group]						
Quant: Study‐specific measure	Attitudes towards older people and interdisciplinary education	*N* = 63 (% unknown) [*N* = 30 nurses *N* = 33 social workers (SW)]	Pre and post‐test survey	Item analysis only: 2 IPE items/21 items significant Item 1 I am willing to take on tasks outside my job description that seem important [nurses *p* = 0.162, NS] [SW *p* < 0.001, +] Item 2 My colleagues from other disciplines are not committed to working together [nurses *p* = 0.07, NS] [SW *p* < 0.001, +]	No (items related to IPE are not related to dementia)	Level 1
Qual: Uni‐professional focus group	n/a	Unclear	Not specified	Theme 1: ‘Differences in professional training, context and role expectations' (SW only) Theme 2: 'Stereotypical views of nurses' personal characteristics' (SW only) Theme 3: ‘Nursing students perceived that social work students had no idea whatsoever about the amount of care involved in caring for a person with dementia, especially in regard to physical/personal care.’ (nurses only) Theme 4: Interprofessional working (both cohorts of students made comments demonstrating seeing the worth in interprofessional working and expressing a need for more understanding)	Yes (theme 3‐Negative perceptions of others' experience)	Level 1
Mastel‐Smith, et al. [28]**** [mixed methods ‐ pre/post survey and focus group]						
Qual: Interprofessional focus group	What went well, what needed improvement, from which activities did they learn new information, what was it about the activity that facilitated learning, barriers to learning, and suggestions for future programs.	*N* = 43	Colaizzi's (1978) seven‐step method guided qualitative data analysis.	Theme 1: Interprofessional education. Students gave positive appraisals of IPL and the authors suggest that students' comments reflected interprofessional competencies, specifically communication and teamwork.	No	Level 1
Balzer, et al. [29]** [pre/post survey]						
Quant: Study specific measure	Survey on course characteristics and, attitudes and knowledge of dementia	*N* = 18 (100%)	Pre/post survey (evaluation of IPE characteristics, post survey only)	Only descriptive for individual items presented items related to IPE: Inter‐professional learning was a great asset to me most frequent response: 72% absolutely agree On reflection: How important do you feel it is that the course will maintain the focus on inter‐professional learning in the future? Most frequent response: 72% ‘important'.	No	Level 1
Chambers, et al. [31]*** [pre/post survey]						
Quant: KidSIM attitude questionnaire	Student perceptions of and attitudes towards IPE, teamwork, and simulation.	*N* = 12 (100%) (Nb. There were 12 control 12 intervention participants, but only the intervention group completed IPE measure)	Pre/Post survey	Total *p* = 0.006 + [relevance of the simulation *p* = 0.058 NA] [Opportunities for IPE *p* = 0.005 +] [Communication *p* = 0.022+ ] [Roles and responsibilities *p* = 0.003 +] [Situation Awareness = 0.039 −] *authors report this as improved.	No	Level 1
Studies with level 2 evidence: Acquisition of knowledge/skills						
Annear, et al. [23]** [pre/post survey]						
Quant: University of West England interprofessional questionnaire (UWEIPQ)	Attitudes towards interprofessional experiences and the educational environment	*N* = 127 (% unknown)	Pre/post‐survey	No total score [communication and teamwork *p* < 0.001, +] [Interprofessional relationships *p* = 0.02, + ] [Interprofessional learning *p* = 0.83, NS ] [Interprofessional interaction *p* = 0.44, NS ]	No	Level 2
Davison, et al. [25]** [post‐intervention survey]						
Qual: 18 open‐text questions in the survey	Students' perceptions of the intervention in relation to how they will care for a person with dementia.	*N* = 60	Thematic analysis	Theme 1: Learning to work together. [Students were able to discuss and share their different perspectives and experiences in interprofessional groups' where they gained a deeper insight into the challenges faced by people with communication difficulties and together explored ways to overcome them’ '' [it was] useful to work in an interprofessional team as practise for the future, helpful exercise in promoting communication.”	No	Level 2
Dressel, et al. [26]*** [pre/post survey]						
Quant: German version of the UWE‐IP (UWE‐IP‐D)	Measure self‐perceived interprofessional attitudes	*N* = 42 (79%)	Pre/post survey	Total score *p* < 0.001, + [communication and teamwork *p* < 0.001, +] [Interprofessional relationships *p* < 0.001 = +] [Interprofessional learning *p* = 0.002 = +] [Interprofessional interaction *p* < 0.001 = +]	No	Level 2
Quant: Study specific measure	Assess self‐reported acquisition of knowledge and skills related to interprofessional teamwork in dementia management, generic knowledge and skills on dementia and patient‐centred dementia care, and interprofessional communication skills	*N* = 42 (79%)	Pre/post survey	Total score *p* < 0.001, + [Domain IP teamwork in dementia management *p* < 0.001, +] [Domain knowledge and skills on dementia care mean score difference *p* < 0.001, +] [Domain IP communication *p* < 0.001, +],	Yes	Level 2
Lawlis, et al. [27]** [mixed methods case study design ‐ pre/post survey						
Quant: A modified version of the readiness for interprofessional learning scale (RIPLS)	Attitudes towards and knowledge of interprofessional learning	*N* = 12 (100%)	Pre‐post survey	Item analysis only: 2/19 items significant Item 1 learning with health‐care students before qualifications would improve relationships after qualifications *p* = 0.028, + Item 2 I learnt a lot from the students from the other disciplines = *p* = 0.046, +	No	Level 1
Qual: Debriefing sessions	‘Themes describing these learning experience, based on data from the debriefing sessions and qualitative components of the survey'. Unclear data sources; debriefing data is notes from educators, not clear what open test questions are.	*N* = 12	Thematic analysis	Theme 1: IPE learning. Subthemes: Teamwork, rewarding, understanding other professionals, communication, and understanding of the environment.	No	Level 2
Brown, et al. [30]*** [pre/post survey]						
Quant: Study specific measure	Satisfaction with the Programme; the perceived value of educational components was measured and the perception of learning outcomes met.	*N* = 196 (67%)	Pre/post survey	Descriptive items presented only: Concerning IPE outcomes, the five learning objectives were individually rated as ‘met’ by 82.8%–92.7% of participants for each cohort. Learning objective 5 (team problem solving) was rated the highest (*M* = 89.8% ‘met’), with the lowest rating for learning objective 4 (team communication) (*M* = 86.6% ‘met’).	No	Level 1
Quant: Readiness for interprofessional learning scale (RIPLS)	Attitudes towards and knowledge of interprofessional learning	*N* = 196 (67%)	Pre/post survey	Total score *p* = 0.30 NS	No	n/a
Quant: Perceived challenges of interprofessional collaboration (PCIC)	Perceived challenges of interprofessional collaboration	*N* = 196 (67%)	Pre/post survey	Total score *p* < 0.0001 +	No	Level 2
Cartwright, et al. [10]** [mixed methods pre/post survey]						
Quant: Interprofessional socialization and valuing scale (ISVS)	Students' interprofessional beliefs, behaviours and attitudes	*N* = 42 (34%)	Pre/post survey	Total score *p* < 0.001 + [ability to work with others *p* < 0.001 +] [Value in working with others *p* < 0.001 +] [Comfort in working with others *p* < 0.002 +]	No	Level 2
Qual: 1 open text question	1. In what ways has the collaboration with other students changed your thinking in terms of client‐centred care and approach to health care?	*N* = 65	Thematic analysis	Theme 1 ‘increased value and understanding of the roles and paradigms of other professions and how team members can work together to meet the needs of their client’ Theme 2 ‘a stronger appreciation of the importance of client‐centred care when working with clients with dementia and complex needs’ Theme 3 ‘students’ appreciation of the importance of negotiating common goals to ensure holistic Management, whilst demonstrating how much students can Learn from each other through shared problem solving’	Yes	Level 1
O'Sullivan, et al. [22]**** [mixed methods pre/post survey]						
Quant: One item included (added to ADKS)	A single item on student awareness of other healthcare professional roles	*N* = 102	Post item only	70% of students felt much more aware of the role of other healthcare professionals after the workshop	No	Level 1
Qual: Open text questions	Open text questions included in the survey (not specified)	*N* = 102	Summative content analysis	Theme 1: Role recognition (sub theme: Communication). ‘Students highlighted the value of interprofessional communication and how this platform gave them an opportunity to communicate with each other as well as discussing what communication strategies would be effective in the case scenario’. Theme 2: Interactive learning (subtheme: Group work/MDT collaboration) ‘students highlighted the value of MDT collaboration and how working together through the case scenario as a team ensured role clarification. The breadth of disciplines represented was valued by students, as were the interesting and differing perspectives offered in discussions around the case study. Some students commented that this was their first experience of MDT collaboration’.	No	Level 2
Studies with level 3 evidence: Behavioural change						
n/a						
Studies with level 4 evidence: Change in organisational practise						
n/a						
Studies with level 5 evidence: 5. Benefits to patients/clients						
n/a						

^a^ based on a modified Barr et al., framework [12]. Modification of attitudes/perceptions (students). Acquisition of knowledge/skills (students). Behavioural change (students). Change in organisational practise (organisation). Benefits to patients/clients (people with dementia/carers).

All studies used a survey, with the majority using a pre/post design (*n* = 10) with a single cross‐sectional design [25]. Only one study had a control group [31] however, the control group did not contribute to IPE outcomes and therefore control group data were not included in the extraction. Six studies incorporated a qualitative component within the survey, this included open‐text survey responses (*n* = 3) [10, 22, 25], focus groups (*n* = 2) [24, 28] and debriefing sessions (*n* = 1) [27].

Study‐specific measures were the most common quantitative outcomes used (*N* = 5) [2, 24, 26, 29, 30]. The Readiness for Interprofessional Learning Scale (RIPLS) [27, 30] and the University of West England Interprofessional Questionnaire (UWE‐IP) [23, 26] were used twice. Other measures used were the Perceived Challenges of Interprofessional Collaboration (PCIC) [30], Interprofessional socialization and valuing scale (ISVS) [10], and the KidSIM Attitude questionnaire [31].

Out of the five outcome levels (based on a modified Barr et al., framework [2, 12]), only two levels were found to have evidence of improvement: attitudes and perceptions towards IPE/IPC (level 1) and knowledge or skills towards IPC (level 2). No studies investigated behavioural change in practise (level 3), or organisational (level 4), or patient outcomes (level 5).

#### 3.3.1 Level 1: Attitudes/Perception

Four interventions found evidence of change in attitudes or perceptions for IPE outcomes.

Dingwall et al., [24] found quantitative and qualitative evidence for a change in student attitudes towards older people and interdisciplinary education, for a novel drama‐based reflective activity ‘Sliding Doors’. Analysis of items on a study‐specific questionnaire found significant improvement on 2/21 items reflecting more positive attitudes to IPC and perceptions of other student groups. However, whilst uni‐professional focus groups described some positive influences (‘differences in professional training, context and role expectations' and value in ‘interprofessional working’) there were also indications of negative attitudes (towards other student groups). The authors conclude that ‘*IPE in its current form will not impact positively on outcomes for older people, unless both professions can openly acknowledge the reality of their professional contexts and develop an understanding of each other's professional restrictions, opportunities and aspirations.*’

Balzer et al., [29] found some indication of increased positive appraisal of IPE, after taking part in Better COMpetencies for the inter‐professional and individually appropriate care for people with DEMentia (KOMPIDEM) programme. However, evidence was limited to descriptive statistics of items on a study‐specific measure. Chambers et al., [31] found that after a short simulation and education module, about delirium focussing on dementia, there were significantly improved student attitudes towards IPE, teamwork, and simulation (KidSIM questionnaire). Lastly, an evaluation of a ‘dementia care bootcamp’ [28] describes some indications of positive appraisal of IPE in qualitative findings, although the description of the findings is limited.

#### 3.3.2 Level 2: Knowledge/Skills

Seven studies found evidence that interventions increased knowledge and skills related to IPE.

An evaluation of the Wicking Dementia Research and Education Centre (WDREC) Teaching Aged Care Facilities (TACF) programme found a significant increase in positive attitudes towards interprofessional relationships, and communication and teamwork (UWEIPQ) [23]. Dressel et al., [26] also found significant increases in scores on the German version of the UWE‐IP (UWE‐IP‐D), on all dimensions (Communication and teamwork, Interprofessional relationships, Interprofessional learning, and Interprofessional interaction) for an interprofessional dementia workshop. In addition, they found a significant increase in a study‐specific measure of knowledge and skills related to interprofessional teamwork in dementia, and skills and communication.

Two more studies found quantitative evidence for an increase in student knowledge and skills. Students taking part in a novel, geriatric, simulation‐enhanced interprofessional education (Gero IPE‐Sim) had a significant reduction in perceived challenges of interprofessional collaboration (PCIC), but no changes in attitudes to IPE (RIPLS) [30]. A study on an online dementia case study [10] found some qualitative evidence of increased attitudes to IPC, as well as significant increases in the ability to work with others, value in working with others, and comfort in working with others (ISVS).

Three studies found qualitative evidence for impact on knowledge or skills. Findings related to IPE were limited as these qualitative analyses explored multiple outcomes. Davison et al., [25] included 18 open‐text questions to evaluate a ‘DA learning package’. Only one theme from the thematic analysis related to IPE: learning to work together. Students described how the IPE helped to develop their communication between student groups. An evaluation of an ‘IPL dementia workshop’ [22] analysed open text responses included in a survey, using summative content analysis. Two themes related to IPE were presented: role recognition and interactive learning. This demonstrated evidence of positive attitudes to IPE as well as some indication of developing communication strategies. A case study evaluation (*n* = 12) [27] of an IPE Residential aged care facilities (RACF) programme found some evidence for increases in attitudes to IPE (RIPLS) and debriefing sessions suggested students' perceived development of IPC knowledge.

#### 3.3.3 Dementia‐Related IPE Outcomes

Three studies described dementia‐specific IPE outcomes. Only one study explicitly identified dementia‐related IPC outcomes and developed a study‐specific measure to assess this [26]. The measure assessed self‐reported acquisition of knowledge and skills related to interprofessional teamwork in dementia management, generic knowledge and skills on dementia and patient‐centred dementia care, and interprofessional communication skills. A significant increase in all subscales was found.

In regards to qualitative findings, themes were less able to be categorised as dementia‐specific due to the level of detail presented; however, themes in two studies were identified. Cartwright et al., 2015 [10] asked students to describe how collaboration changed their thinking about care practices. Themes included dementia‐specific IPC skills, including ‘negotiating common goals to ensure holistic management’ and learning ‘how team members can work together to meet the needs of their client’. Dingwall et al., [24] identified a negative finding: after the intervention, nursing students perceived social work students as not having realistic expectations about the level of care for a person with dementia.

### 3.4 Risk of Bias

Out of a score of five (highest quality), two studies were rated 4, three were rated 3, and six were rated 2 (shown in Table 3). A consistent limitation was no control or comparator groups, or not accounting for potential confounders in the analysis. There were limitations in reporting the numbers of participants at baseline, including no clear distinction between those taking part in the intervention versus those taking part in the evaluation. Qualitative components that were included as part of the survey often lacked a description of methods.

## 4 Discussion

The main aim of this review was to examine the evidence about whether interprofessional dementia education for undergraduate healthcare students positively impacts outcomes related to collaborative practise. The results indicate that there is some evidence that IPE dementia educational interventions may increase student attitudes towards IPE and IPC (level 1), and some evidence of perceived impacts on knowledge or skills or IPC (level 2). There was no evidence for student behavioural change or organisational or patient outcomes (levels 3–5).

### 4.1 Evidence for IPE Outcomes

The findings suggest some increase in the evidence base over the past 10 years compared with the previous systematic review [9] but IPE educational interventions and dementia interventions continue to mostly evaluate lower‐level outcomes [2, 16]. One study that recognised this limitation investigated behaviour (level 3) by surveying students at a later time point when in practise [22]. However, outcomes presented were limited to dementia skills (communication modification with dementia patients), not IPC skills. Future studies of dementia educational interventions need to evaluate behavioural outcomes in students for generic and dementia‐specific IPC as well as dementia skills when an educational intervention has an IPE component.

Overall, most of the studies' primary focus was on evaluating dementia attitudes and knowledge outcomes rather than outcomes related to IPC. For example, Mastel‐Smith et al., [28] included measures of dementia knowledge, attitudes, confidence and empathy and found significant improvements, but only provided limited exploration of IPC outcomes in qualitative findings. Whilst the main learning outcomes of these dementia education interventions may be centred on dementia attitudes and knowledge, the inclusion of an IPE element suggests an expectation of influence on IPC competencies. Therefore, the evaluation of IPC outcomes needs more consideration, as it is important to understand the benefit of running education in interprofessional groups. This is especially important when delivering content inter‐professionally, which requires additional complexity, for example, timetable fit and collaboration between departments, as well as pedagogic considerations to ensure content is relevant to students of different professions.

Furthermore, papers often did not describe the planned learning outcomes of interventions or what the specific IPE learning outcomes were. Future studies should specify student learning outcomes (identified during intervention design [2]), and ensure these align with measured outcomes. Interventional outcomes for IPC should be guided by work on interprofessional competency frameworks [32, 33, 34].

In terms of the strength of evidence (for types of intervention), the varying and generally low quality of evaluations prevented comparisons and conclusions on optimal modalities. However, in general, studies demonstrated positive evidence to suggest that dementia IPE may have the potential to improve student attitudes and knowledge of IPC. The most frequent interventions were CBL, simulation, or multiple modalities. This is consistent with the literature on IPE in general, where positive outcomes are found for a range of teaching methods, but lacks consensus on the most effective teaching approaches [14]. Research on dementia education suggests that direct exposure to people with dementia is important for developing positive attitudes and a person‐centred approach [16]. Further research is needed to understand the components of effective IPE interventions in the context of dementia care.

### 4.2 Evidence for Dementia‐Specific IPE Outcomes

Dementia care appears to be an increasing context for IPE education; 11 interventions were identified, and dementia has been cited as a frequent subject of IPE interventions in a systematic review of IPE interventions [2]. The evidence presented in this review continues to suggest that dementia care is a suitable context for IPE learning. Dementia IPE may have the dual benefit of promoting generic as well as dementia‐specific IPC skills, which is identified as requiring improvement in clinical practise [8]. However, out of 11 included studies, only three explored dementia‐specific IPC outcomes. All quantitative measures of IPE outcomes were generic except one, a study‐specific measure that has not been validated. The development of validated dementia IPC measures would allow a better understanding of dementia‐specific IPC learning.

## 5 Limitations

The conclusion that dementia IPE interventions have a positive impact on student IPC should be interpreted with caution due to the design of the included studies, which, consistent with many evaluations of educational interventions, were pragmatic and quasi‐experimental. A considerable limitation is that no studies included a control group, therefore introducing a risk of bias. However, whilst positive publication bias may be a factor, all studies presented some (if not definitive) evidence to support positive IPE outcomes. Limitations introduced by the review process include the exclusion of languages other than English. Overall, this review provides a comprehensive overview of educational literature on dementia IPE in the last decade and can inform curriculum developers and future research.

## 6 Conclusion

This review demonstrates an increase in evidence that interprofessional dementia education for undergraduate healthcare students can positively impact outcomes related to collaborative practise. These data suggest that IPE components of dementia educational interventions may contribute to IPC competencies, as well as dementia skills. However, methodological quality in studies to date was generally low, with outcomes limited to student attitudes and perceived knowledge. More studies of higher methodological quality are needed that investigate higher‐level outcomes and IPC learning outcomes, including generic and dementia‐related interprofessional competencies.

## Funding

This work was supported by NHS England. NHS England had no role in the design or completion of this study.

## Conflicts of Interest

The authors declare no conflicts of interest.

## Supporting information


Supporting Information S1

Supporting Information S2

Supporting Information S3

## Data Availability

The data that support the findings of this study are available from the corresponding author upon reasonable request.
