# Supplementary material for: Interprofessional Dementia Education in Pre‐Registration Healthcare Students: A Systematic Review
Source: Int J Geriatr Psychiatry. 2026 May 16;41:e70213. doi: 10.1002/gps.70213 (PMC13179845; doi:10.1002/gps.70213)
Supplement: Supplementary file 2 — Supporting Information S2 [file GPS-41-e70213-s002.pdf]

		**25/06/2024**
**Ovid (uob)**	EMBASE	**519**
**PROQUEST (sussex)**	APA PsycInfo®	**133**
	ASSIA (PROQUEST)	**81**
	ProQuest Dissertations & Theses Global	**27**
**Ebsco (uob)**	British Education Index;	**2**
	ERIC	**5**
	CINAHL	**204**
	Medline	**265**
**COCHRAINE LIBRARY.**	n/a	**98**
**wos**		**340**
**All references**		**1674**
**Duplicate's removed**		**850**

Search Name: 18062023 IPE
Date Run: 25/06/2024 16:14:16
Comment:

ID Search Hits
#1 MeSH descriptor: [Interprofessional Education] this term only 11
#2 MeSH descriptor: [Interdisciplinary Placement] this term only 10
#3 interdisciplin* or inter-disciplin* or interprofession* or inter-profession* or multiprofession* or multi-profession*:ti,ab 5493
#4 student* or undergraduate or pre-registration or preregistration or baccalaureate or train* or education*:ti,ab 258252
#5 MeSH descriptor: [Education] explode all trees 45666
#6 demen* or alzheimer*:ti,ab 35281
#7 MeSH descriptor: [Dementia] explode all trees 9444
#8 #1 or #2 or #3 5493
#9 #4 or #5 273804
#10 #6 or #7 35728
#11 #8 AND #9 AND #10 with Publication Year from 2014 to 2024, in Trials 98

#	Query	Limiters/Expanders	Last Run Via	Results
S4	S1 AND S2 AND S3	Expanders - Apply equivalent subjects<br>Search modes - Proximity	Interface - EBSCOhost Research Databases<br>Search Screen - Basic Search<br>Database - British Education Index	2
S3	TI (demen* or alzheimer*) OR AB (demen* or alzheimer*) OR SU dementia	Expanders - Apply equivalent subjects<br>Search modes - Proximity	Interface - EBSCOhost Research Databases<br>Search Screen - Basic Search<br>Database - British Education Index	303
S2	TI (student* or undergraduate or pre-registration or preregistration or baccalaureate or train* or education*) OR AB (student* or undergraduate or pre-registration or preregistration or baccalaureate or train* or education*) OR DE education OR SU education	Expanders - Apply equivalent subjects<br>Search modes - Proximity	Interface - EBSCOhost Research Databases<br>Search Screen - Basic Search<br>Database - British Education Index	282,798
S1	TI (interdisciplin* or inter-disciplin* or interprofession* or inter-profession* or multiprofession* or multi-profession*) OR AB (interdisciplin* or inter-disciplin* or interprofession* or inter-profession* or multiprofession* or multi-profession*) OR SU 'interprofessional education' OR DE	Expanders - Apply equivalent subjects<br>Search modes - Proximity	Interface - EBSCOhost Research Databases<br>Search Screen - Basic Search<br>Database - British Education Index	2,440

'interprofessional
education'

EBSCOhost

Tue, June 25, 2024 03:10:40 pm

#	Query	Limiters/Expanders	Last Run Via	Results
S4	S1 AND S2 AND S3	Limiters - Publication Date: 20140101-20241231<br>Expanders - Apply equivalent subjects<br>Search modes - Proximity	Interface - EBSCOhost Research Databases<br>Search Screen - Basic Search<br>Database - CINAHL Plus	204
S3	TI (demen* or alzheimer*) OR AB (demen* or alzheimer*) OR MH Dementia OR SU dementia	Expanders - Apply equivalent subjects<br>Search modes - Proximity	Interface - EBSCOhost Research Databases<br>Search Screen - Advanced Search<br>Database - MEDLINE	310,712
S2	TI (student* or undergraduate or pre-registration or preregistration or baccalaureate or train* or education*) OR AB (student* or undergraduate or pre-registration or preregistration or baccalaureate or train* or education*) OR (SU education) OR (MH "Health Education")OR (MH "Health Education") OR (MH "Education, Health Sciences")	Expanders - Apply equivalent subjects<br>Search modes - Proximity	Interface - EBSCOhost Research Databases<br>Search Screen - Basic Search<br>Database - CINAHL Plus	1,100,034
S1	TI (interdisciplin* or inter-disciplin* or interprofession* or inter-profession* or multiprofession* or multi-profession*) OR AB (interdisciplin* or inter-disciplin* or interprofession* or inter-profession* or	Expanders - Apply equivalent subjects<br>Search modes - Proximity	Interface - EBSCOhost Research Databases<br>Search Screen - Advanced Search<br>Database - MEDLINE	73,309

	multiprofession* or multi-profession*) OR (MH "Interprofessional Education") OR (MH "Interdisciplinary Placement") OR SU 'interprofessional education'	

25/06/2024

Embase Classic+Embase <1947 to 2024 June 24>

1 (interdisciplin* or inter-disciplin* or interprofession* or inter-profession* or multiprofession* or multi-profession*).ti,ab. or *interprofessional education/ 100862

2 (student* or Undergraduate or pre-registration or preregistration or baccalaureate or train* or education*).ti,ab. or *education/ 2253811

3 (demen* or alzheimer*).ti,ab. or *dementia/ 410193

4 1 and 2 and 3 671

5 limit 4 to yr="2014 - 2024" 519

EBSCOhost

#	Query	Limiters/Expanders	Last Run Via	Results
S4	S1 AND S2 AND S3	Limiters - Published Date: 20140101-20241231<br>Expanders - Apply equivalent subjects<br>Search modes - Proximity	Interface - EBSCOhost Research Databases<br>Search Screen - Basic Search<br>Database - ERIC	5
S3	TI (demen* or alzheimer*) OR AB (demen* or alzheimer*) OR DE dementia	Expanders - Apply equivalent subjects<br>Search modes - Proximity	Interface - EBSCOhost Research Databases<br>Search Screen - Basic Search<br>Database - ERIC	1,583
S2	TI (student* or undergraduate or pre-registration or preregistration or baccalaureate or train* or education*) OR AB (student* or undergraduate or pre-registration or preregistration or baccalaureate or train* or education*) OR DE education	Expanders - Apply equivalent subjects<br>Search modes - Proximity	Interface - EBSCOhost Research Databases<br>Search Screen - Basic Search<br>Database - ERIC	1,299,249
S1	TI (interdisciplin* or inter-disciplin* or interprofession* or inter-profession* or multiprofession* or multi-profession*) OR AB (interdisciplin* or inter-disciplin* or interprofession* or inter-profession* or multiprofession* or multi-profession*) OR DE	Expanders - Apply equivalent subjects<br>Search modes - Proximity	Interface - EBSCOhost Research Databases<br>Search Screen - Basic Search<br>Database - ERIC	35,789

"Interdisciplinary
Approach"

#	Query	Limiters/Expanders	Last Run Via	Results
S4	S1 AND S2 AND S3	Limiters - Publication Date: 20140101-20241231<br>Expanders - Apply equivalent subjects<br>Search modes - Proximity	Interface - EBSCOhost Research Databases<br>Search Screen - Advanced Search<br>Database - MEDLINE	265
S3	TI (demen* or alzheimer*) OR AB (demen* or alzheimer*) OR MH Dementia OR SU dementia	Expanders - Apply equivalent subjects<br>Search modes - Proximity	Interface - EBSCOhost Research Databases<br>Search Screen - Advanced Search<br>Database - MEDLINE	310,712
S2	TI (student* or undergraduate or pre-registration or preregistration or baccalaureate or train* or education*) OR AB (student* or undergraduate or pre-registration or preregistration or baccalaureate or train* or education*) OR (MH "Education")	Expanders - Apply equivalent subjects<br>Search modes - Proximity	Interface - EBSCOhost Research Databases<br>Search Screen - Advanced Search<br>Database - MEDLINE	1,663,849
S1	TI (interdisciplin* or inter-disciplin* or interprofession* or inter-profession* or multiprofession* or multi-profession*) OR AB (interdisciplin* or inter-disciplin* or interprofession* or inter-profession* or multiprofession* or multi-profession*) OR (MH "Interprofessional Education") OR (MH	Expanders - Apply equivalent subjects<br>Search modes - Proximity	Interface - EBSCOhost Research Databases<br>Search Screen - Advanced Search<br>Database - MEDLINE	73,309

"Interdisciplinary
Placement") OR SU
'interprofessional
education'

Search Strategy from ProQuest

25 June 2024 08:18

SEARCH STRATEGY

Set No.	Searched for	Databases	Results
S1	(tiab(interdisciplin* OR interprofession* OR inter-profession* OR inter-disciplin* OR multiprofession* OR multi-profession*.) OR sub('interprofessional education')) AND (tiab(Dement* OR Alzheimer*) OR sub(dementia)) AND (tiab(student* OR undergraduate OR pre-registration OR preregistration OR baccalaureate OR train* OR education) OR sub(education)) AND pd(>20131231)	APA PsycInfo®	133

Database copyright © 2024 ProQuest LLC. All rights reserved.

Terms and Conditions Contact ProQuest

Search Strategy from ProQuest

25 June 2024 08:56

SEARCH STRATEGY

Set No.	Searched for	Databases	Results
S12	((tiab(interdisciplin* OR interprofession* OR inter-profession* OR inter-disciplin* OR multiprofession* OR multi-profession*.) OR sub('interprofessional education') OR MAINSUBJECT.EXACT.EXPLODE("Interdisciplinary education") OR MAINSUBJECT.EXACT.EXPLODE("Interdisciplinary aspects") OR MAINSUBJECT.EXACT.EXPLODE("Interprofessional cooperation")) AND (tiab(Dement* OR Alzheimer*) OR sub(dementia)) AND (tiab(student* OR undergraduate OR pre-registration OR preregistration OR baccalaureate OR train* OR education) OR sub(education)) AND pd(>20131231))	Applied Social Sciences Index & Abstracts (ASSIA)	81

Database copyright © 2024 ProQuest LLC. All rights reserved.

Terms and Conditions Contact ProQuest

Search Strategy from ProQuest

25 June 2024 08:39

SEARCH STRATEGY

Set No.	Searched for	Databases	Results
S9	((tiab(interdisciplin* OR interprofession* OR inter-profession* OR inter-disciplin* OR multiprofession* OR multi-profession*.) OR sub('interprofessional education')) AND (tiab(Dement* OR Alzheimer*) OR sub(dementia)) AND (tiab(student* OR undergraduate OR pre-registration OR preregistration OR baccalaureate OR train* OR education) OR sub(education)) AND pd(>20131231))	ProQuest Dissertations & Theses Global	27

Database copyright © 2024 ProQuest LLC. All rights reserved.

Terms and Conditions   Contact ProQuest

Web of Science Search Strategy (v0.1)

Database: Web of Science Core Collection

Entitlements:

- WOS.IC: 1993 to 2024
- WOS.CCR: 1985 to 2024
- WOS.SCI: 1900 to 2024
- WOS.AHCI: 1975 to 2024
- WOS.BHCI: 2005 to 2024
- WOS.BSCI: 2005 to 2024
- WOS.ESCI: 2005 to 2024
- WOS.ISTP: 1990 to 2024
- WOS.SSCI: 1900 to 2024
- WOS.ISSHP: 1990 to 2024

Searches:

1: (TI=(interdisciplin* OR inter-disciplin* OR interprofession* OR inter-profession*OR multiprofession* OR multi-profession*) OR AB=(interdisciplin* OR inter-disciplin* OR interprofession* OR inter-profession*OR multiprofession* OR multi-profession*) OR TS=('interprofessional education')) NOT (SILOID==("PPRN")) Date Run: Tue Jun 25 2024 14:44:08 GMT+0100 (British Summer Time) Results: 146348

2: TI=(student* OR undergraduate OR pre-registration OR preregistration OR baccalaureate OR train* OR education*) OR AB=(student* OR undergraduate OR pre-registration OR preregistration OR baccalaureate OR train* OR education*) OR TS=(education) NOT (SILOID==("PPRN")) Date Run: Tue Jun 25 2024 14:45:46 GMT+0100 (British Summer Time) Results: 3866049

3: (TI=(Dement* OR Alzheimer*) OR AB=(Dement* OR Alzheimer*)) OR TS=(dementia) NOT (SILOID==("PPRN")) Date Run: Tue Jun 25 2024 14:48:37 GMT+0100 (British Summer Time) Results: 382938

4: #3 AND #2 AND #1 Timespan: 2014-01-01 to 2024-12-31 Date Run: Tue Jun 25 2024 14:49:08 GMT+0100 (British Summer Time) Results: 340
