# Supplementary material for: Interprofessional Dementia Education in Pre‐Registration Healthcare Students: A Systematic Review
Source: Int J Geriatr Psychiatry. 2026 May 16;41:e70213. doi: 10.1002/gps.70213 (PMC13179845; doi:10.1002/gps.70213)
Supplement: Supplementary file 3 — Supporting Information S3 [file GPS-41-e70213-s003.docx]

**APPENDIX C**

Screening procedure for titles and abstracts using AS Review; based on SAFE procedure for stopping criterion (Boetje & van de Schoot, 2024).

PHASE 1 Pre-screen random subset **[Researchers: MH, SM, PR, YF]**

Complete together as a training exercise on selected random papers stratified by year of publication. Using screening template- adjust as needed.

- Screen a minimum of **[ 1% ]** of the total number of papers. **[N=9]**

(or more up until). Find at least one relevant record. **[22 exclude 1 include]**

- Calculate estimate for relevant records= **[1/23*850 = 37]**

PHASE 2 Active learning (complete set) **[MH & SM]**

Complete in duplicate and compare.

- A minimum of **[30%]** of the total dataset has been screened **[= 255]**

[NB. Increased estimate due to sample]

- All key papers have been marked as relevant;
- No extra relevant records have been identified in the last **50** records
- At least twice the estimate of the number of relevant records **[=74 records]** have been screened [NB. caution due to relatively small size: may be over conservative, inspect the recall plot in instances where a large number of consecutive records to see if plateau reached]

PHASE 3 Find more using deep learning (complete labelled set) **[MH]**

Reload data file with labels from previous phases.

- Use a different model. **[Model used: Random forest and Sbert]**
- No extra relevant records are identified in the last **[50]** records. **[1 identified, 66 further screened, 321 total screened]**

PHASE 4 Evaluate quality (all records labelled as irrelevant) **[MH]**

- Import the 10 highest (Relevant) and lowest-ranked papers(irrelevant) from phase 2, all papers previously marked irrelevant. No extra relevant records are identified in the last **[50]** records. **[None found]**
- Further quality checks at next stage: Citation checks for more papers.
